# Comparisons of constitutive resistances to soybean cyst nematode between PI 88788- and Peking-type sources of resistance in soybean by transcriptomic and metabolomic profilings

**DOI:** 10.3389/fgene.2022.1055867

**Published:** 2022-11-10

**Authors:** Shanshan Chu, Hui Ma, Ke Li, Junfeng Li, Hongli Liu, Leipo Quan, Xuling Zhu, Meiling Chen, Wenyan Lu, Xiaoming Chen, Xuelian Qu, Jiaqi Xu, Yun Lian, Weiguo Lu, Erhui Xiong, Yongqing Jiao

**Affiliations:** ^1^ Collaborative Innovation Center of Henan Grain Crops /College of Agronomy, Henan Agricultural University, Zhengzhou, China; ^2^ Zhengzhou Subcenter of National Soybean Improvement Center, Key Laboratory of Oil Crops in Huang-Huai Valleys of Ministry of Agriculture, Institute of Industrial Crops, Henan Academy of Agricultural Sciences, Zhengzhou, China

**Keywords:** soybean, soybean cyst nematode, Peking, PI 88788, transcriptome, metabolome

## Abstract

Soybean cyst nematode (SCN) is a serious damaging disease in soybean worldwide. Peking- and PI 88788-type sources of resistance are two most important germplasm used in breeding resistant soybean cultivars against this disease. However, until now, no comparisons of constitutive resistances to soybean cyst nematode between these two types of sources had been conducted, probably due to the influences of different backgrounds. In this study, we used pooled-sample analysis strategy to minimize the influence of different backgrounds and directly compared the molecular mechanisms underlying constitutive resistance to soybean cyst nematode between these two types of sources *via* transcriptomic and metabolomic profilings. Six resistant soybean accessions that have identical haplotypes as Peking at *Rgh1* and *Rhg4* loci were pooled to represent Peking-type sources. The PI88788-type and control pools were also constructed in a same way. Through transcriptomic and metabolomics anaylses, differentially expressed genes and metabolites were identified. The molecular pathways involved in the metabolism of toxic metabolites were predicted to play important roles in conferring soybean cyst nematode resistance to soybean. Functions of two resistant candidate genes were confirmed by hairy roots transformation methods in soybean. Our studies can be helpful for soybean scientists to further learn about the molecular mechanism of resistance to soybean cyst nematode in soybean.

## Introduction

Soybean (*Glycine max*) is an important oil and protein crop worldwide. However, soybean cyst nematode (SCN), a widely spread pathogen, is one of major threats to soybean production (Niblack et al., 2006). In addition to crop rotation, planting resistant cultivars is the most efficient and practical method to control this pest. To date, many sources of resistance to SCN have been found in soybean (Concibido et al., 2004). Among them, Peking, PI 88788, PI 437654 and PI 209332 are four widely used germplasm in breeding SCN-resistant cultivars.

To reveal genetic basis of resistance for these sources, quantitative trait locus (QTL) analyses were performed (Wang et al., 2001; Concibido et al., 2004; Vuong et al., 2010). Among them, two QTLs, *Rhg1* and *Rhg4*, were found to play the most important roles in conferring SCN resistance soybean (Concibido et al., 2004). *Rhg1* was located on Chromosome 18 (Chr. 18) (Concibido et al., 2004). This locus was found in almost all of reported SCN-resistant sources, except for PI 567516C (*Glycine max*) and one wild soybean line PI 468916 (*Glycine soja*) (Wang et al., 2001; Concibido et al., 2004; Vuong et al., 2010). There were two different alleles at the *Rhg1* locus, *Rhg1*-a from Peking and *Rhg1*-b from PI 88788 (Concibido et al., 2004; Kim M. et al., 2010). These two alleles might have different mechanisms of resistance to SCN. *Rhg1*-a has to interact with *Rhg4* in conferring resistance to SCN HG type 0 (race 3), while *Rhg1*-b is able to function independently (Meksem et al., 2001; Concibido et al., 2004). Moreover, Peking- and PI 88788-type sources showed different responses to SCN infections (Kim Y. H. et al., 2010). PI 437654, one of Peking-type sources, showed a rapid resistance reaction, which was characterized by the death of SCN at the parasitic second (J2) stage juvenile (Kim Y. H. et al., 2010). In contrast, PI 88788 has a prolonged resistance reaction where nematode die at the J3 and J4 stage (Kim Y. H. et al., 2010). *Rhg1*-b had been cloned from PI 88788 through map-based cloning (Cook et al., 2012). Three genes, *Glyma.18g022400* (*Glyma18g02580*), *Glyma.18g022500* (*Glyma18g02590*) and *Glyma.18g022700* (*Glyma18g02610*) in together, were found to be responsible for this locus. The resistance to SCN in PI 88788 was attributed to an elevated level of constitutive expressions for these three genes, which were caused by increased copy numbers rather than sequence mutation (Cook et al., 2012). Further studies showed that *Rhg1*-a in Peking contained specific SNPs, around half of copies, and different levels of methylations for these three genes in comparison with *Rhg1*-b in PI 88788 (Cook et al., 2014; Liu et al., 2017). For *Rhg4*, it was identified to be a serine hydroxymethyltransferase (SHMT) that was involved in cellular one-carbon metabolism (Liu et al., 2012). In Peking, gain of function mutations that resulted in amino acid changes, altered the regulatory property of the Rhg4 protein and conferred new functions to this gene (Liu et al., 2012).

Scientists made great efforts to understand molecular mechanisms of interaction between soybean and SCN. Gene expression studies on either compatible or incompatible responses to SCN were comprehensively conducted through a serial of methods including differential display of mRNA (Hermsmeier et al., 1998), cDNA microarray (Khan et al., 2004; Alkharouf et al., 2006), affymetrix Genechip (Klink et al., 2007b; Ithal et al., 2007), and laser capture micro-dissection (LCM) (Klink et al., 2007a). And, interactions between soybean and SCN were also investigated by identifying and studying the effectors that SCN secreted during infection (Bellafiore and Briggs, 2010; Gheysen and Mitchum, 2011). All these studies suggested that the a complicated network composed of pathways of auxin, cell wall, secondary metabolites, programed cell death and defense responses, was involved in the interaction between soybean and SCN (Gheysen and Mitchum, 2011).

Currently, almost all reported SCN-resistant germplasms were either Peking- or PI 88788-type (Concibido et al., 2004; Cook et al., 2014; Jiao et al., 2015). Peking-type sources were characterized by containing both *Rhg1*-a and *Rhg4*, while PI 88788-type sources of resistance were characterized by containing *Rhg1*-b (Cook et al., 2014; Kadam et al., 2016). Different SCN-resistant genes as well as different reactions in response to SCN infection suggested different molecular mechanisms of resistance to SCN between these two types of sources. However, no direct comparisons between these two types of resistant sources have been reported until now. Thus, the common and different characteristics of resistance mechanism between these two resistant sources remain unknown. This might due to the influence of different backgrounds between these two germplasm and resulted difficulty to obtain accurate information.

In this studies, to investigate characteristics of constitutive resistance mechanism between Peking and PI 88788-type sources of resistance and overcome influences of different backgrounds, we used pooled-sample analysis strategy to conduct transcriptomic and metabolomic studies for these two types of resistant sources. The common and specific genes and metabolites were identified. The possible resistance mechanisms between these two sources were also put forward. Our results could be helpful for scientists to further learn about the molecular mechanisms underlying resistance to SCN in soybean.

## Results

### Pools of soybean accessions based on genotypic and phenotypic data

Based on the genomic and phenotypic data (Kadam et al., 2016), three pools of soybean accessions were constructed, which included: (Pool-I) Peking-type sources containing PI 548402 (Peking), PI 90763, PI 404166, PI 89772, PI 437654 and PI 438489B, which have same haplotypes as Peking at *Rhg1* and *Rhg4* loci; (Pool-II) PI 88788-type sources containing PI 88788, PI 437655, PI 495017C, PI 209332, PI 438503 A and PI 467312, which have same haplotypes as PI 88788 at *Rhg1* locus but do not contain *Rhg4*; and (Pool-III) non-resistant controls PI 518664 (Hutcheson), PI 518671 (Williams 82) and PI 595362 (Magellan), which do not contain *Rhg1* and *Rhg4* (Table 1). Evaluations of SCN resistances revealed that the resistant accessions in Pool-I exhibited resistance or moderately resistance to SCN HG type 2.5.7, 0 and 2.5.7, while the accessions in Pool-II showed resistant or moderately resistant to SCN HG type 0 and 1.3.5.6.7. Both Pool-I and Pool-II showed resistance to SCN HG type 0 in common (Table 1).

**TABLE 1:** Bulks of Peking- and PI 88788-type sources of resistance to soybean cyst nematode (SCN).

PIs	*Rhg1*	*Rhg4*	FI (%) of SCN HG type (SCN isolate)				
						**2.5.7**	**1.2.5.7**	**0**	**2.5.7**	**1.3.5.6.7**
PI 548402	+	+	R (2.1)	S (72.3)	R (2.6)	MR (18.6)	S (69.6)
PI 90763	+	+	R (9.2)	R (9.6)	R (1.8)	R (4.5)	MS(43.9)
PI 404166	+	+	R (3.6)	MS(30.7)	R (3.8)	MR (11.0)	MS(41.0)
PI 89772	+	+	R (3.2)	MR (14.7)	R (3.5)	R (10.0)	MS(49.9)
PI 437654	+	+	R (1.9)	R (1.7)	R (1.4)	R (7.2)	R (9.5)
PI 438489B	+	+	R (1.1)	MR (11.4)	R (2.7)	MR (13.3)	MR (15.7)
PI 88788	++	−	MS(42.1)	MS(44.4)	R (8.1)	MS(59.0)	R (8.4)
PI 437655	++	−	MR (28.6)	MR (26.2)	R (4.4)	MS(38.3)	R (5.5)
PI 495017C	++	−	MR (29.8)	MR (22.8)	R (5.9)	MS(51.7)	MR (14.1)
PI 209332	++	−	MS(39.1)	MS(41.5)	R (6.4)	MS(49.8)	MR (21.7)
PI 438503 A	++	−	MS(49.8)	S (61.4)	R (6.3)	S (82.7)	MR (18.6)
PI 467312	++	−	MS(46.9)	MS(52.3)	R (8.8)	MS(32.5)	MR (15.6)

+ represents the existence of and *Rhg4* or low copies of *Rgh1* in the genome. ++ represent the existence of high copies of *Rhg1* in the genome. − represents inexistence of *Rhg4* in the genome. Represents the missing data. Female index (FI) (%) values are calculated from three replicates. R resistant, FI < 10; MR, moderately resistant, 10 < FI < 30; MS, moderately susceptible, 30 < FI < 60; S susceptible, FI > 60.

### Transcriptomic analyses for two types of sources of resistance to soybean cyst nematode

**FIGURE 1:**
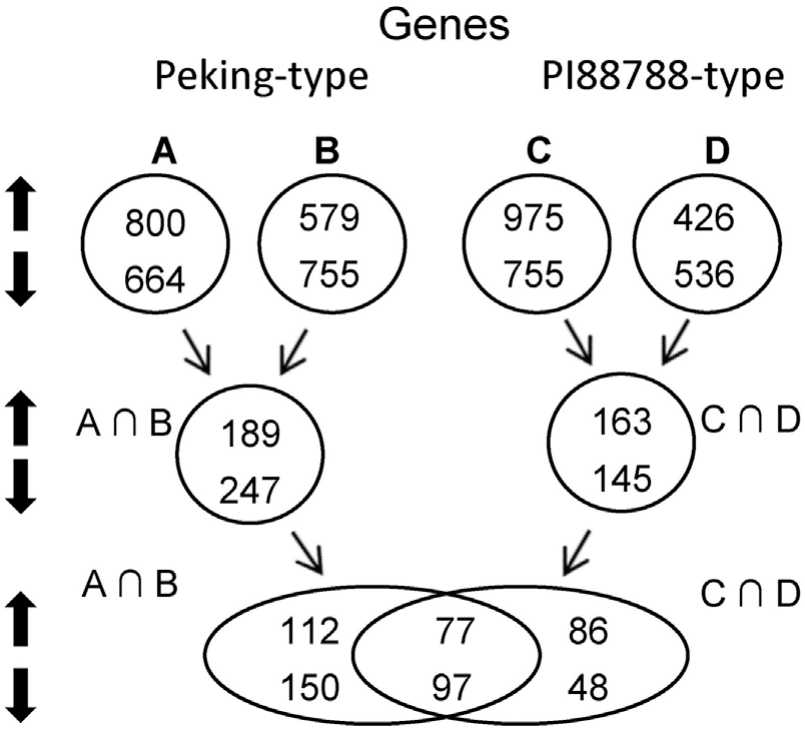
Strategy of pooled-sample analysis and the number of significant differentially expressed genes (DEGs) between Peking- and PI 88788-type sources of resistance. A, Peking *versus* (vs.) Hutcheson; B, Pool-a vs. Pool-c: the pool of PI 90763, PI 404166, PI 89772, PI 437654, and PI 438489B vs. the pool of Williams 82 and Magellan; C, PI 88788 vs. Hutcheson; D, Pool-b vs. Pool-c: the pool of PI 437655, PI 495017C, PI 209332, PI 438503A and PI 467312 vs. the pool of Williams 82 and Magellan; A∩B, intersection between **(A)** and **(B)**; C∩D, intersection between **(C)** and **(D)**.

To investigate the differences of constitutive resistance between these two types of sources of resistance, we conducted transcriptomic profilings for these accessions. The root samples of Pool-I were divided into two groups, Peking and the pool of PI 90763, PI 404166, PI 89772, PI 437654, and PI 438489B (Pool-a). The root samples of Pool-II were divided into PI 88788 and the pool of PI 437655, PI 495017C, PI 209332, PI 438503 A and PI 467312 (Pool-b). And, the root samples of Pool-III were divided into Hutcheson and the pool of Williams 82 and Magellan (Pool-c). Two comparisons for Pool-I were conducted, which included Peking *versus* (vs.) Hutcheson and Pool-a vs. Pool-c (Figure 1; Supplementary Tables S1, 2). To minimize the influences of different backgrounds, genes with same expression patterns between these two comparisons, were chosen to represent the gene expression patterns of Peking-type sources (Pool-I) (Figure 1; Supplementary Table S3). Similarly, two comparisons including PI 88788 vs. Hutcheson and Pool-b vs. Pool-c were also conducted (Figure 1; Supplementary Tables S4, 5). And, genes with same expression patterns between these two comparisons were chosen to represent the gene expression patterns of PI 88788-type sources (Pool-II) (Figure 1; Supplementary Table S6).

In Peking-type sources, a total of 436 significantly differentially expressed genes (DEGs) were identified, where 189 were up-regulated and 247 were down-regulated (Figure 1; Supplementary Table S3). In PI 88788-type sources, a total of 308 DEGs were identified, where 112 were up-regulated and 150 were down-regulated (Figure 1; Supplementary Table S6). Among these DEGs, 112 up-regulated and 150 down-regulated genes were specific for Peking-type sources (Figure 1; Supplementary Table S7) while 86 up-regulated and 48 down-regulated genes were specific for PI 88788-type sources (Figure 1; Supplementary Table S8). Seventy-seven up-regulated and 97 down-regulated genes were commonly found in both of these two types of sources (Figure 1; Supplementary Table S9). Expressions of three responsible genes at *Rhg1*, Glyma.18g022400, Glyma.18g022500 and Glyma.18g022700 as internal standards were up-regulated in accordance with copy number variation of *Rhg1* in Peking- and PI 887788- type sources (Cook et al., 2012) (Cook et al., 2014; Liu et al., 2017), which could prove the reliability of our transcriptomic profilings (Supplementary Table S9).

GO and KEGG analyses were conducted for these DEGs (Table 2). In Peking-type sources, the specifically up-regulated DEGs were significantly enriched in the biological processes including fatty acid biosynthetic process (GO:0006633), monocarboxylic acid biosynthetic process (GO:0072330), and inositol metabolic process (GO:0006020), and molecular pathways including Linoleic acid metabolism (ko00591), benzoxazinoid biosynthesis (ko00402), flavone and flavonol biosynthesis (ko00944), and plant-pathogen interaction (ko04626) (Table 2), while the specifically down-regulated DEGs were significantly enriched in the biological processes including response to stimulus (GO:0050896), oxazole or thiazole biosynthetic process (GO:0018131), oxazole or thiazole metabolic process (GO:0046484), and molecular pathways including plant-pathogen interaction (ko04626) (Table 2). In PI 88788-type sources, no significant biological processes and pathways were detected for the specific DEGs (Table 2). In both of these two sources, the common up-regulated DEGs were significantly enriched in the carotene metabolic process (GO: 0016119), while the common down-regulated DEGs were significantly enriched in the plant-pathogen interaction pathway (ko0462) (Table 2). Interestingly, the plant-pathogen interaction pathway (ko0462) was commonly detected for both up- and down-specifically regulated genes in Peking-type sources, indicating different specific pathways might be affected by these genes.

**TABLE 2:** The biological process ontology and pathway analysis of differentially expressed genes (DEGs) between Peking- and PI 88788- type sources of resistance (Pool-I and Pool-II).

DEGs	Patterns	GO ID	Annotation	*p* Value	KO ID	Annotation	Q value
Peking-type specific	Up –regulated	GO:0006633	fatty acid biosynthetic process	0.02	ko00591	Linoleic acid metabolism	0.009
		GO:0072330	monocarboxylic acid biosynthetic process	0.03	ko00402	Benzoxazinoid biosynthesis	0.04
		GO:0006020	inositol metabolic process	0.04	ko00944	Flavone and flavonol biosynthesis	0.04
					ko04626	Plant-pathogen interaction	0.04
	Down-regulated	GO:0050896	response to stimulus	0.001	ko04626	Plant-pathogen interaction	0.0003
		GO:0018131	oxazole or thiazole biosynthetic process	0.03			
		GO:0046484	oxazole or thiazole metabolic process	0.03			
common	Up –regulated	GO:0016119	carotene metabolic process	0.04	NA	NA	NA
	Down-regulated	NA	NA	NA	ko04626	Plant-pathogen interaction	0.001
PI 88788-type specific	Up –regulated	NA	NA	NA	NA	NA	NA
	Down-regulated	NA	NA	NA	NA	NA	NA

Pool-I, PI 548402 (Peking), PI 90763, PI 404166, PI 89772, PI 437654 and PI 438489B. Pool-II, PI 88788, PI 437655, PI 495017C, PI 209332, PI 438503 A and PI 467312. NA, none.

In addition to general analyses, we also investigated the functions of these DEGs in detail. Because a large number of DEGs were involved in plant secondary metabolism, we selected some interesting genes that might be involved in producing toxic or antioxidant secondary metabolites and conferring constitutive SCN resistance to soybean (Table 3). Four predicted O-methyltransferase encoding genes that participate in the conversion of TRIBOA-glucoside to DIMBOA-glucoside in the last step of benzoxazinoid biosynthesis pathway (ko00402) were selected, among which Glyma.06G286600, Glyma.09G094600 and Glyma.06G286200 were specifically up-regulated in Peking-type sources and Glyma.09G094400 was commonly up-regulated in both of two resources (Table 3; Supplementary Figure S1). Two genes, Glyma.09G144900 and Glyma.09G145000, were specifically up-regulated in PI 88788-type sources, which were predicted to encode a 2-oxoglutarate (2OG) and Fe(II)-dependent oxygenase respectively and might be involved in conversion of DIBOA-glucoside to TRIBOA-glucoside, the penultimate step of benzoxazinoid biosynthesis (Table 3; Supplementary Figure S1). The natural benzoxazinoids could function as pesticides and insecticides (Barnes and Putnam, 1987) (Grombacer AW and Guthrie, 1989) (Bravo and Lazo, 1993) (Sicker et al., 2000). The DIBOA biosynthesis is regarded as the core pathway for benzoxazinoids. DIMBOA is produced by the modification of DIBOA and has more reactive activity than DIBOA. In plants, benzoxazinoids are mainly stored in vacuole as glucosides (Sicker et al., 2000). Upon cell damage, the benzoxazinone glucosides is released from the vacuole and produced the toxic aglucone by a specific glucosidase (Esen, 1992). Up-regulation of these genes might indicate the enhancement of benzoxazinoid biosynthesis pathway and DIMBOA-glucosite content in these resistance sources, especially in Peking-type sources. Except for genes in the benzoxazinoid pathway, several genes in cyanoamino acid metabolism were also selected, which included *Glyma.15G031400*, *Glyma.18G080400*, *Glyma.08G150100* and *Glyma.15G270900* (Table 3; Supplementary Figure S2). Cyanohydrins in plants are found usually in the form of cyanogydrin glycosides, which was relatively stable and stored in the plant cell vacuole (Flematti et al., 2013). Cyanohydrins can be hydrolyzed by a hydroxynitrile lyase to release cyanide, which was the toxic metabolites and confer defense against herbivores and pathogens (Flematti et al., 2013). Three up-regulated DEGs, *Glyma.15G031400*, *Glyma.08G150100*, and *Glyma.15G270900* were Peking-type specific, common, and PI 88788-type specific, respectively. All of these three genes were predicted to encode β-glucosidases and participated in conversion of cyanoglycoside to cyanohydrin (ko00460) (Table 3; Supplementary Figure S2). The *Glyma.18G080400* was a Peking-type specific down-regulated DEG, which was predicted to be a cytochrome P450 and involved in conversion of aldoxime to cyanohydrin (ko00460) (Table 3; Supplementary Figure S2). These results indicated that the branch from amygdalin to cyanohydrin in cyanoamino acid metabolism might be enhanced in both Peking-type and PI 88788-type sources, while the branch from l-amino acid to cyanohydrin might be specifically reduced in Peking-type resistant sources (ko00460).

**TABLE 3:** The selected differentially expressed genes (DEGs) related to metabolites involved in constitutive resistances of Peking- and PI 8878- type sources of resistance (Pool-I and Pool-II).

DEGs	Expression	Gene ID	Predicted funciton	Pathway	KO
Peking-type specific	Up-regulated	Glyma.06G286600	O-methyltransferase family protein	Benzoxazinoid biosynthesis; Phenylpropanoid biosynthesis	ko00402; ko00940
		Glyma.09G094600	O-methyltransferase family protein		
		Glyma.06G286200	O-methyltransferase family protein	Benzoxazinoid biosynthesis	ko00402
		Glyma.15G031400	BGLU15|beta glucosidase 15	Cyanoamino acid metabolism; Phenylpropanoid biosynthesis	ko00460; ko00940
		Glyma.07G196800	LOX3|lipoxygenase 3	Alpha-linolenic acid metabolism	ko00592
		Glyma.04G131100	isoflavone reductase	Flavonoid biosynthesis	ko00941
		Glyma.02G130400	CHS, TT4, ATCHS|Chalcone and stilbene synthase family protein		
	Down-regulated	Glyma.18G061300	2-oxoglutarate (2OG) and Fe(II)-dependent oxygenase superfamily protein	Cysteine and methionine metabolism	ko00270
		Glyma.18G080400	CYP71B34|cytochrome P450, family 71, subfamily B, polypeptide 34	Cyanoamino acid metabolism; Phenylpropanoid biosynthesis; Isoflavonoid biosynthesis	ko00460; ko00940; ko00943
		Glyma.11G164700	NAD(P)-binding Rossmann-fold superfamily protein	Phenylpropanoid biosynthesis	ko00940
		Glyma.18G055400	Peroxidase superfamily protein		
		Glyma.08G101000	HXXXD-type acyl-transferase family protein	PHENYLPROPANOID biosynthesis; Flavonoid biosynthesis	ko00940; ko00941
		Glyma.08G140500	TT7, CYP75B1, D501|Cytochrome P450 superfamily protein	PHENYLPROPANOID biosynthesis	ko00940
		Glyma.08G109300	CHS, TT4, ATCHS|Chalcone and stilbene synthase family protein	Flavonoid biosynthesis	ko00941
		Glyma.07G083000	CYP76C4|cytochrome P450, family 76, subfamily C, polypeptide 4		
Common	Up-regulated	Glyma.09G094400	O-methyltransferase family protein	Benzoxazinoid biosynthesis; Phenylpropanoid biosynthesis	ko00402; ko00940
		Glyma.08G150100	BGLU12|beta glucosidase 12	Cyanoamino acid metabolism; Phenylpropanoid biosynthesis	ko00460; ko00940
		Glyma.07G204800	alpha/beta-Hydrolases superfamily protein	Alpha-linolenic acid metabolism	ko00592
		Glyma.10G209800	PAL2, ATPAL2|phenylalanine ammonia-lyase 2	Phenylpropanoid biosynthesis	ko00940
		Glyma.02G239400	AAE5|acyl activating enzyme 5		
		Glyma.10G200800	CYP76C4|cytochrome P450, family 76, subfamily C, polypeptide 4	Flavonoid biosynthesis; Isoflavonoid biosynthesis	ko00941; ko00943
		Glyma.02G048600	F3H, TT6, F3′H|flavanone 3-hydroxylase		
	Down-regulated	Glyma.05G147000	CCOAMT|caffeoyl-CoA 3-O-methyltransferase	Phenylpropanoid biosynthesisi; Flavonoid biosynthesis	ko00940; ko00941
		Glyma.U000100	CYP76C4|cytochrome P450, family 76, subfamily C, polypeptide 4	Flavonoid biosynthesis	ko00941
PI 88788-type specific	Up-regulated	Glyma.09G144900	2-oxoglutarate (2OG) and Fe(II)-dependent oxygenase superfamily protein	Cysteine and methionine metabolism; Benzoxazinoid biosynthesis	ko00270; ko00402
		Glyma.09G145000	2-oxoglutarate (2OG) and Fe(II)-dependent oxygenase superfamily protein	Benzoxazinoid biosynthesis; Flavonoid biosynthesis	ko00402; ko00941
		Glyma.15G270900	BGLU17|beta glucosidase 17	Cyanoamino acid metabolism; Phenylpropanoid biosynthesis	ko00460; ko00940
		Glyma.15G245300	CYP72A15|cytochrome P450, family 72, subfamily A, polypeptide 15	Brassinosteroid biosynthesis	ko00905
		Glyma.07G089900	CYP71A22|cytochrome P450, family 71, subfamily A, polypeptide 22	Phenylpropanoid biosynthesis	ko00940
		Glyma.05G021800	TT7, CYP75B1, D501|Cytochrome P450 superfamily protein	Flavonoid biosynthesis	ko00941
		Glyma.13G068800	CYP82C4|cytochrome P450, family 82, subfamily C, polypeptide 4		
	Down-regulated	Glyma.09G277800	Peroxidase superfamily protein	Phenylpropanoid biosynthesis	ko00940
		Glyma.01G130800	Peroxidase superfamily protein		
		Glyma.15G203500	CYP82C4|cytochrome P450, family 82, subfamily C, polypeptide 4	Flavonoid biosynthesis	ko00941

Pool-I, PI 548402 (Peking), PI 90763, PI 404166, PI 89772, PI 437654 and PI 438489B. Pool-II, PI 88788, PI 437655, PI 495017C, PI 209332, PI 438503A and PI 467312.

### Metabolomic analyses for two types of sources of resistance to soybean cyst nematode

To reveal the differences of metabolism between Peking-type and PI 88788-type sources, we conducted and compared metabolomic profilings by use of LC-MS for the same samples that were used for transcriptomic profilings.

**FIGURE 2:**
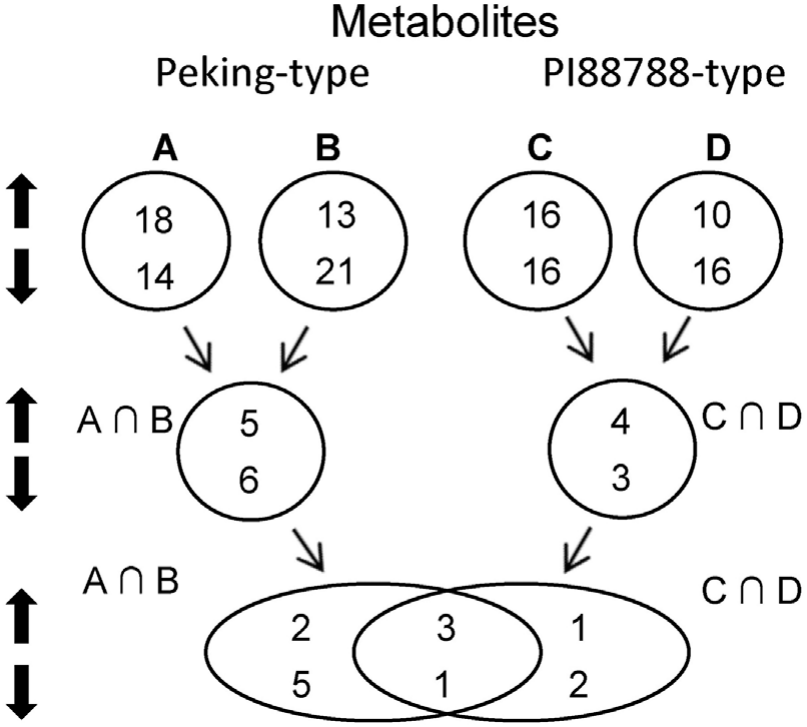
Bulk strategy and number of significant differentially expressed metabolites (DEMs) between Peking- and PI 88788-type sources of resistance. A, Peking *versus* (vs.) Hutcheson; B, Pool-a vs. Pool-c: the pool of PI 90763, PI 404166, PI 89772, PI 437654, and PI 438489B vs. the pool of Williams 82 and Magellan; C, PI 88788 vs. Hutcheson; D, Pool-b vs. Pool-c: the pool of PI 437655, PI 495017C, PI 209332, PI 438503A and PI 467312 vs. the pool of Williams 82 and Magellan; A∩B, intersection between **(A)** and **(B)**; C∩D, intersection between **(C)** and **(D)**.

To minimize the influences of different backgrounds, the same strategy of pooled-sample analysis was adopted for the analyses of metabolism as that in the transcriptome analysis (Figure 2; Supplementary Table S10). In comparison with the controls (Pool-III), five up-regulated metabolites including HOME, jasmonic acid, kamlolenic acid, S-adenosylmethionine and traumatic acid, and six down-regulated ones including cGMP, aminooctanoic acid, glycitein-O-glucuronide, HpOTrE, hydroxy-octadecadiynoic acid and linolenic acid, were significantly detected for Peking-type sources (Table 4). KEGG pathway analyses showed that the up-regulated metabolites were mainly involved in the pathway of biosynthesis of plant hormones (ko01070), and α-linolenic acid metabolism (ko00592) (Table 4). In comparison, the down-regulated metabolites were mainly involved in pathways of purine metabolism (ko00230), arginine and proline metabolism (ko00943),α-Linolenic acid metabolism (ko00592), and linoleic acid metabolism (ko00591) (Table 4). Two pathways of arginine and proline metabolism (ko00943) and α-Linolenic acid metabolism (ko00592) were detected for both of up- and down-regulated metabolites (Table 4). For PI 88788-type sources, four up-regulated metabolites including HOME, jasmonic acid, quercetin and traumatic acid, and three down-regulated ones including aminooctanoic acid, phenylalanine and TriHOME, were significantly identified (Table 4). KEGG Pathway analyses showed that the up-regulated metabolites were mainly involved in the pathways of α-linolenic acid metabolism (ko00592) and flavonoid biosynthesis (ko00941) (Table 4). In comparison, the down-regulated metabolites were mainly involved in the pathway of phenylalanine, tyrosine and tryptophan biosynthesis (ko00400), and linoleic acid metabolism (ko00591) (Table 4). No common pathways were detected for both up- and down-regulated genes (Table 4). By comparing the changes of metabolites between Peking-type and PI 88788-type sources, we found that three up-regulated metabolites including HOME, jasmonic acid and traumatic acid, and one down-regulated metabolite, aminooctanoic acid, were commonly detected for both of these two comparisons (Table 4). Jasmonic acid and traumatic acid were both involved in the pathways of α-linolenic acid metabolism (ko00592) (Table 4).

**TABLE 4:** Significant differentially expressed metabolites (DEMs) between Peking- and PI 88788-type sources of resistance.

DEMs	Pattern	Metabolite	Compound	Pathway
Peking-type	Up-regulated	HOME	C08365	NA
		Jasmonic acid	C08491	alpha-Linolenic acid metabolism
		kamlolenic acid	C16342	alpha-Linolenic acid metabolism
		S-Adenosylmethionine	C00019	Ethylene biosynthesis
		Traumatic Acid	C16308	alpha-Linolenic acid metabolism
	Down -regulated	cGMP	C00942	Purine metabolism
		Aminooctanoic acid	NA	NA
		Glycitein 7-O-glucoside	C16195	Isoflavonoid biosynthesis
		HpOTrE	C16321	alpha-Linolenic acid metabolism
		hydroxy-octadecadiynoic acid	C14762	Linoleic acid metabolism
		Linolenic Acid	C06427	alpha-Linolenic acid metabolism
PI 88788-type	Up-regulated	HOME	C08365	NA
		Jasmonic acid	C08491	alpha-Linolenic acid metabolism
		Quercetin	C00389	Flavonoid biosynthesis
		Traumatic Acid	C16308	alpha-Linolenic acid metabolism
	Down-regulated	Aminooctanoic acid	NA	NA
		Phenylalanine	C00079	Phenylalanine, tyrosine and tryptophan biosynthesis
		TriHOME	C14833	Linoleic acid metabolism

Pool-I, PI 548402 (Peking), PI 90763, PI 404166, PI 89772, PI 437654 and PI 438489B. Pool-II, PI 88788, PI 437655, PI 495017C, PI 209332, PI 438503A and PI 467312. NA, not available.

Combined with transcriptome data, we found that, one gene, Glyma.07G204800, in α-Linolenic acid metabolism (KO00592) was commonly up-regulated in both of Peking-type and PI 88788-type sources (Table 3). This gene was predicted to encode an α/β -Hydrolase, which catalyzed phosphatidylcholine to α-Linolenic acid (ko00592) (Supplementary Figure S3). Up-regulation of Glyma.07G204800 might activate the production of α-Linolenic acid and enhance the content of relevant metabolites such as traumatic acid and jasmonic acid, which was proved by the metabolome data (Table 4). The metabolites analyses showed that the phenylalaninine was specifically down-regulated in PI 88788-type sources (Table 4). Accordingly, *Glyma.06G235900* that encodes an aminotransferase was also specifically down-regulated in PI 88788-type sources, which was involved in conversion of phenylpyruvate to phenylalanine (ko00400) (Supplementary Figure S4).

### Functional confirmation of candidate resistant genes

**FIGURE 3:**
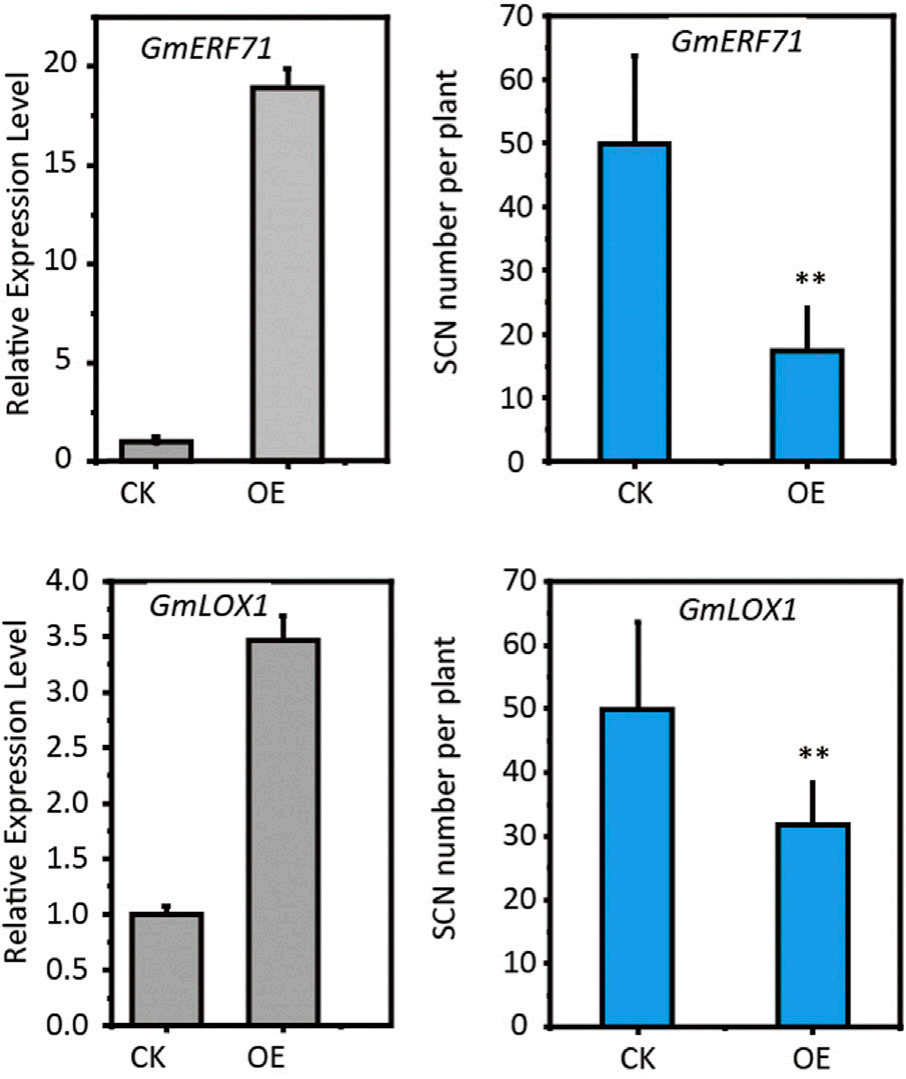
Functional confirmation of *GmERF71* and *GmLOX1*. CK, control; OE, plants with gene overexpressed. The significance was determined by *t* test. *n* = 10.

To investigate the effectiveness of our strategy, we selected two candidate resistant genes, *GmERF71* (*Glyma.19G262700*) and *GmLOX1* (*Glyma.15G026500*), to confirm their functions in conferring SCN resistance to soybean through hairy-root transformation methods. The *GmERF71*encodes an AP2 protein and *GmLOX1* encodes a lipoxygenase. Both of these two genes were up-regulated in Peking-type and PI88788-type sources of resistance (Supplementary Tables S3 and S6). We generated transgenic hairy roots by overexpressing these two genes under 35S promoter (Supplementary Figure S5). The qRT-PCR results confirmed the increased expression levels of *GmERF71* and *GmLOX1* (Figure 3), confirming the success of our hairy root transformation. The evaluation of SCN resistance for transgenic hairy roots showed overexpression of these two genes could significantly decrease the cyst number on hairy roots (Figure 3), confirming their functions in conferring SCN resistance to soybean.

## Disscussion

### Rationality of our comparisons of constitutive resistance between Peking- and PI 88788-type sources of resistance

For *Rhg1*-b in PI 88788, about ten copies of three responsible genes, Glyma.18g022400, Glyma.18g022500 and Glyma.18g022700 cause constitutively up-regulated expression of these three genes and as a result the changes of constitutive molecular pathways *in vivo* (Cook et al., 2012). In Peking, despite around four copies of these three genes were found for *Rhg1*-a, their expressions were also constitutively enhanced (Cook et al., 2012; Cook et al., 2014; Kadam et al., 2016). In addition, for *Rhg4* in Peking, the alteration of gene function rather than loss of function could cause the alterations of constitutive metabolisms *in vivo* either (Liu et al., 2012). These constitutive changes were proved to play important roles in conferring SCN resistance to soybean (Liu et al., 2012; Cook et al., 2014). Our summary of phenotypic data showed that Peking-type accessions was resistant or moderately resistant to SCN HG types 2.5.7, 0 and 2.5.7, while PI 88788-type accessions were resistant or moderately resistant to SCN HG types 0 and 1.3.5.6.7 (Table 1). Both of them were resistant to SCN HG type 0. These results indicated these two types of resistance sources might have common and different characteristics of mechanisms underlying constitutive resistances. To reveal these characteristics will provide important information for scientists to elucidate the resistance mechanisms for Peking and PI88788-type resistant germplasm. Unfortunately, until now, little has been known about constitutive resistances for these two types of sources of resistance. That might be due to the influences of different backgrounds between these two sources and as a result no direct comparisons could be conducted. To overcome this problem, we developed and used the pooled-sample analysis strategy. Six resistant soybean accessions that have haplotypes identical to Peking at *Rhg1* and *Rhg4*, were selected to constitute the Peking-type pool (Kadam et al., 2016). Similarly, the PI88788-type pool was also constructed. Theoretically, these two pools only have differences at *Rhg1* and Rhg4 *loci*. Thus, the direct comparisons of SCN resistances between these two types of sources of resistances could be realized. The genes and metabolites associated with constitutive SCN resistances underlying these two types of sources were identified in our studies, suggesting the rationality of our strategy.

### The mechanisms of constitutive resistance to soybean cyst nematode revealed by transcriptomic analyses

Since both Peking- and PI 88788-type sources were resistant to SCN HG type 0, there might be a common resistant mechanism shared by these two types of resistance sources. Seventy-seven up-regulated and 97 down-regulated DEGs were commonly identified in both of these two sources. The analyses of GO and KEGG pathways revealed that the defense response (GO: 0006952), plant-pathogen interaction (ko04626), linoleic acid metabolism (ko00591), isoflavonoid biosynthesis (ko00943), phenylpropanoid biosynthesis (ko00940) etc, were significantly detected for common DEGs (Table 2). These molecular pathways were reported to be associated with resistance to abiotic and biotic stresses in plants (Hernandez et al., 2009; Suzuki et al., 2014). In addition to genes selected in Table 3, others are also worthy of being studied in the further. For example, two lipoxygenase 1-enconding genes, *Glyma.15G026500* and *Glyma.04G105900* were found to be up-regulated in both Peking- and PI 88788-type sources (Supplementary Table S9). Lipoxygenase is an important enzyme in the biosynthesis of jasmonic acid that is an important wounding-related phytohormone involved in resistance to biotic stresses (Dar et al., 2015; Carvalhais et al., 2017). In the process of SCN infection, the J2 SCN juveniles need enter into soybean roots and continue moving until they found the loci where the syntium can be established (Gheysen and Mitchum, 2011). During this process, the wounding to plants by SCN happened continuously. The enhancement of JA content might enable soybean to fight against this infection wounding and consequently confer SCN resistance to soybean.

Except for the common resistance to SCN HG type 0, Peking- and PI 88788- type resistant sources showed specific resistance phenotypes, where Peking-type sources are resistant or moderately resistant to SCN HG type 2.5.7 and HG type 2.5.7 while PI 88788-type sources are resistant or moderately resistant to HG type 1.3.5.6.7 (Table 1). These specific phenotypes indicated that there might be specific resistance mechanisms underlying these two types of sources. In order to gain insights into specific mechanisms, we also investigated specific DEGs between Peking- and PI 88788- type resistant sources (Supplementary Tables S7, 8). Four significant pathways including Linoleic acid metabolism (ko00591), benzoxazinoid biosynthesis (ko00402), Flavone and flavonol biosynthesis (ko00944), Plant-pathogen interaction (ko04626), were specific for Peking-type sources, which indicated that these pathways might play important roles in conferring specific SCN resistance to Peking-type sources (Table 2). In PI 88788-type sources, no significant pathways were detected (Table 2). However, a large number of DEGs that were involved in defense signal transductions were detected such as five leucine-rich repeat transmembrane protein kinase (LRR) proteins (*Glyma.12G123100*, *Glyma.15G021400*, *Glyma.15G021700*,*Glyma.16G174100* and *Glyma.16G215000*) and four receptor like proteins (*Glyma.20G118400*, *Glyma.16G176600*, *Glyma.16G176900* and *Glyma.16G175100*) (Supplementary Table S8). Based on the above, we speculated that, in Peking-type sources, defense-related metabolites might play more important roles in response to SCN. The constitutive high level of anti-pathogen/insect metabolites in Peking-type sources could lead to a rapid defense response to the infection of J2 stage nematode (Klink et al., 2011). In contrast, in PI 88788-types sources, defense signaling pathway might be more important. It might take a while for anti-pathogen/insect metabolites to reach a certain level that could influence SCN. This might be the reason why PI 88788-type sources could exhibit a delayed defense response to J3 stage nematode (Klink et al., 2011). Our results might provide clues to reveal the mechanism underlying phenotypic differences between these two resistant germplasm.

### The mechanisms of constitutive resistance to soybean cyst nematode revealed by metabolomic analysis

To investigate the metabolic differences between Peking- and PI 88788-type germplasm, we used the samples identical to the ones for transcriptomic analyses to conduct metabolomic analyses. For Peking-type sources, five up-regulated and six down-regulated metabolites were identified, which were involved in five different pathways including α-Linolenic acid metabolism (ko00592), biosynthesis of plant hormones (ko01070), Purine metabolism (ko00230), Isoflavonoid biosynthesis (ko00943) and Linoleic acid metabolism (ko00591). For PI 88788-type sources, four up-regulated and three down-regulated metabolites were identified, which were involved in six different pathways including α-Linolenic acid metabolism (ko00592), flavonoid biosynthesis (ko00941), phenylalanine metabolism (ko00360) and linoleic acid metabolism (ko00591). Three up-regulated metabolites including HOME, jasmonic acid and traumatic acid, and one down-regulated metabolite, aminooctanoic acid, were commonly identified in both Peking- and PI 88788-type sources (Table 4). HOME is one component of cutin, which is the polymer matrices for lipophilic cell wall barriers and play important roles in protecting plants form biotic and abiotic stresses (Pollard et al., 2008). Jasmonic acid is well known as a defense plant hormone (Dar et al., 2015). Previous studies showed JA pathway played important roles in conferring SCN resistance to soybean in both of PI 88788- and Peking-types sources of resistance (Klink et al., 2009) (Klink et al., 2010; Matsye et al., 2010). Traumatic Acid was one of major products derived from linolenic acid in the hydroperoxidelyase pathway, where a series of important signals in wounding and plant–insect communication were identified (Croft et al., 1993; Kallenbach et al., 2011). The common up-regulation of these three metabolites indicated the enhancement of molecular pathways associated with biotic stresses in both of PI 88788- and Peking-type sources, which might be the reason why both of these two types of sources displayed resistance to SCN HG type 0 in common.

The S-adenosylmethionine that takes part in ethylene and polyamine biosynthesis, were specifically up-regulated in Peking type sources (Table 4). And, glycitein-O-glucuronide that takes part in isoflavonoid biosynthesis was specifically down-regulated (Table 4). Ethylene, polyamine and isoflavonoids were reported to be important metabolites associated with plant-insect interactions (Simmonds, 2003; Hussain et al., 2011; Erb et al., 2012). The specific changes of these metabolites indicated that the ethylene, polyamine and isoflavonoids related pathways might be involved in determining specific phenotypes of Peking-type sources. In PI 88788-type sources, quercetin that takes parts in flavonoid biosynthesis was specifically up-regulated, while phenylalanine that takes parts in phenylalanine, tyrosine and tryptophan biosynthesis, was specifically down-regulated (Table 3). The flavonoids metabolites were largely involved in all aspects of plant life including plant-insect interaction (Ferreyra et al., 2012). Tryptophan is the precursor of auxin, which is an important phytohormone regulating plant development and other processes (Park, 2007) (Zhao, 2010). The changes of these specific metabolites might have important functions in determining specific phenotypes of PI 88788-type sources.

Interestingly, we noticed that some metabolites that belong to the same metabolism pathway were regulated in different patterns in same type of resistant sources. For example, jasmonic acid, kamlolenic acid, and traumatic acid, were up-regulated in Peking-type sources, while HpOTrE and linolenic Acid were down-regulated. All of these metabolites were in the same pathway of α-Linolenic acid metabolism (ko00592). We further investigated the map of α-Linolenic acid metabolism (Supplementary Figure S3) and found that jasmonic acid, kamlolenic acid, traumatic acid and HpOTrE were located in the different branches of α-Linolenic acid metabolism. These results indicated that different branches of same metabolism pathway might be regulated differently in response to SCN, which were worthy of being studied further.

### Integrative analyses of transcriptome and metabolome

Since both transcriptome and metabolome were conducted in this study, we further combined these two analyses and investigated the changes of molecular pathways between Peking- and PI 88788- type sources. The results indicated the transcriptome and metabolome data exhibited good consistency. Lipoxygenase was a key enzyme in the biosynthesis of jasmonic acid, which used linoleate and α-linolenate as substrates (Schaller and Stintzi, 2009). Two putative lipoxygenase 1-encoding genes, *Glyma.15G026500* and *Glyma.04G105900*, were commonly up-regulated in expression in both of Peking- and PI 88788- type sources (Supplementary Table S9). Up-regulation of these two lipoxygenase genes was in accordance with the enhancement of JA content in metabolite profiling (Table 4). In PI 88788-type sources, one intermediate product in flavonoid biosynthesis, quercetin, was specifically up-regulated in content (Table 4). Correspondingly, *Glyma.05G021800* that encodes a putative cytochrome P450 and was involved in flavonoid biosynthesis (ko00941), was also up-regulated (Supplementary Table S8). All these information could provide us useful clues to understand molecular mechanisms underlying constitutive resistance to SCN between these two types of resistance sources. However, due to limited capability of technology for metabolomics analysis, we are not able to get a full and accurate map of metabolites *in vivo* in plants. Thus, changes of lots of metabolites in our comparisons were unavailable. In addition, mRNA level and metabolites are not directly related with each other. Many processes such as gene translation, protein modification, and metabolites stability can affect the consistency between mRNA and metabolite. Thus, in some cases, changes of gene expressions might not reflect actual changes of metabolites.

### Functional confirmation of candidate resistant genes

The *APETALA2* (*AP2*)/*ETHYLENE-RESPONSIVE FACTOR* (*ERF*) is the second largest transcription factor family in plants and plays a important role in stress responses (Feng et al., 2020). The SCN candidate gene *GmERF71* was both up-regulated in Peking-type and PI88788-type sources of resistance. The cyst number on the transgenic hairy roots overexpressing *GmERF71* decreased by 59% compared with the control, confirming its resistant function (Figure 3). Previous studies showed that, in transgenic soybean plants overexpressing *GmERF5* and *GmERF113* the expression of defense related genes such as PR10, PR1-1 and PR10-1 were significantly induced, thereby improving the resistance to soybean *phytophthora blight* (Dong et al., 2015; Zhao et al., 2017). Overexpression of GmERF3 genes in transgenic tobacco also activates the expression of PR genes, enhancing its resistance to pathogens (Zhang et al., 2009). AtERF1 has been proved to act downstream of ethylene and jasmonic acid pathways, and is a key factor in the integration of these two signals to regulate defense response genes (Lorenzo et al., 2003). OsERF3 mediates the biosynthesis of salicylic acid, jasmonic acid, ethylene and hydrogen peroxide through MAPK cascade and WRKY genes, enhancing the resistance of plants to stinging or chewing insects (Lu et al., 2011). We speculate that *GmERF71* participates in the regulation of resistance to SCN by activating downstream defense genes and phytohormone pathways, which need to be studied further.

The lipoxygenase is a key enzyme in the biosynthesis of jasmonic acid (Dar et al., 2015; Wasternack and Song, 2017). It has been reported that lipoxygenase genes are up-regulated in syncytium after SCN infection in Peking and PI88788 (Klink et al., 2009; Klink et al., 2010). Overexpression of three JA biosynthesis genes, *AtAOS*, *AtAOC* and *AtJAR* in soybeans improves the resistance to SCN (Matthews et al., 2014). The *GmLOX1* (*Glyma.15G026500*) were commonly up-regulated in both of Peking- and PI 88788- type sources and were also identified by integrative analyses of transcriptome and metabolome, which were involved in JA biosynthesis. Our results showed that *GmLOX1* overexpression could enhance resistance to SCN in transgenic hairy roots, confirming the role of JA in response to SCN infection.

In summary, we directly and systemically compared the constitutive resistances between Peking- and PI 88788- type sources of resistance in this study, results of which might be helpful for scientists to learn the resistance mechanisms for these two types of sources further.

## Methods

### Plant materials

The soybean accessions used in this study were collected from Germplasm Resources Information Network (GRIN), United States. Seeds were pre-germinated on moistened filter paper in a plant growth chamber at 27°C, 85% ambient humidity and a 16:8 (light: dark) photoperiod for 3–4 days. The seedlings were then transferred into tubes (5 cm 3 30 cm) with sandy soil at 27°C under 16 h of light in the greenhouse. When the plants had three fully expanded trifoliate leaves, roots were harvested, flash frozen in liquid nitrogen, ground to powder, and stored at −80 C until the next step. For each sample, three biological replicates were set up.

### Soybean cyst nematode phenotyping

The SCN assays were performed in greenhouse according to the reported methods (Niblack et al., 2009). In brief, soybean seeds were germinated and transplanted into PVC tubes. The tubes were filled with steam-pasteurized sandy soil and packed into plastic containers prior to transplanting. Five plants of each indicator line and RIL were arranged in a randomized complete block design. Two days after transplanting, each plant was inoculated with about 2000 SCN eggs. Thirty days post-inoculation, nematode cysts were washed from the roots of each plant and counted. The SCN resistances were evaluated by SCN reaction to a set of soybean indicator lines for HG type tests (Peking, PI 88788, PI 90763, PI 437654, PI 209332, PI 89772, PI 548316, and susceptible checks (cv. Hutcheson). The female index (FI %) was estimated to evaluate the response of each plant to each HG type of SCN using the following formula:

FI (%) = (Number of female cyst nematodes on a given individual/average number of female nematodes on the susceptible check) × 100.

### RNA-seq analysis

The root samples were grinded into two parts, one of which was used to extract total RNAs using TRIZOL reagent (Invitrogen, CA, United States) following the manufacturer’s procedure. The total RNA concentration and purity were assayed with a NanoDrop ND-1000 spectrophotometer (NanoDrop Technologies, Wilmington, DE, United States). The RNA integrity was assessed on an Agilent 2,100 Bioanalyzer Lab-on-Chip system (Agilent Technologies, Palo Alto, CA, United States). The construction of cDNA libraries was according to the protocol for the mRNA-Seq sample preparation kit (Illumina, San Diego, United States). The clustering of samples was performed on a cBot Cluster Generation System using TruSeq PE Cluster Kit v3-cBot-HS (Illumia, San Diego, United States) according to the manufacturer’s instructions. And the paired-end sequencing was performed on the Illumina Hiseq2500 platform. Sequencing-received raw image data were transformed by base calling into raw sequences. The raw sequences were transformed into clean tags by removing the three adaptor sequences and low quality tags. All clean tags were mapped to the reference sequences (https://www.soybase.org/), and those tags with no more than one mismatch were considered. The differentially expressed genes (DEGs) with fold change of log2 ≧2 were deemed as significant DEGs. Gene Ontology (GO) enrichment and KEGG pathway analyses for these significant DEGs were carried out in the website of Soybase (https://www.soybase.org/) and KEGG (https://www.genome.jp/kegg/).

### Metabolites analysis

The other root samples were used for LC-MS analysis. Fifty mg of root powders were extracted with 800 μL methanol. After vortex-mixing for 30 s, these samples were centrifuged at 12,000 rpm for 10 min to remove proteins. The supernatant was then transferred to autosampler vials. Metabolic Profiling Analysis of Plasma by LC-Q-TOF MS. Plasma samples for metabolomics assays were thawed at room temperature for about 15 min and vortexed for 5 s. Afterwards, 300 μL methanol of HPLC grade (Merck) was added to 100 μL plasma. After shaking for 30 s and standing for 20 min at 4°C, the mixture was centrifuged at 12,000 rpm for 15 min at 4 C. The supernatant was transferred into a vial, and 4 μL sample was injected into LC-Q-TOF MS (Agilent, 1,290 Infinity LC, 6530 UHD and Accurate-Mass Q-TOF/MS). The plasma extract was injected into a C18 column (Agilent, 100 × 2.1 mm, 1.8 μm) in line with LC system and equilibrated with the mobile phase (A: water containing 0.1% formic acid; B: acetonitrile containing 0.1% formic acid). Samples were eluted by 95% A and 5% B at a flow rate of 0.4 ml/min for 16 min, and 5% A and 95% B at a flow rate of 0.4 ml/min for 3 min. The metabolites were separated by LC, analyzed, and assigned by Q-TOF-MS (Agilent). The Q-TOF was operated in ESI-positive mode.

The capillary and sampling cone voltages were set at 4 kV and 35 kV, respectively. The desolvation gas flow was set to 600 L/h at a temperature of 350°C, and the source temperature was set to 100 °C. The TOF-MS data were collected in the range of m/z 50–1,000 with a scan time of 0.03 s and inter scan time of 0.02 s. In order to insure the accuracy and repeatability, the leucine-enkephalin was applied as lock mass. The changed metabolites with *p* value ≦ 0.05 and FDR ≦0.05 were deemed as significantly changed metabolites. The KEGG pathway analyses for differentially changed metabolites were carried out in the website of KEGG (https://www.genome.jp/kegg/).

### Soybean hairy root transformation

Soybean seeds were sterilized with chlorine gas for 12–14 h and then germinated in Hogland medium for 4 days under 16 h/8 h light/dark conditions in a growth chamber at 25–26 C. Germinating seedlings were used for hairy root transformation as described previously with *Agrobacterium rhizogenes* strain K599 (Kereszt et al., 2007). Transgenic composite plants and controls (cv. Tianlong 2) were transplanted to pots (10 × 10 cm) containing sterilized sands and grown for 3 days. About 2000 SCN eggs were inoculated for each container. Thirty days after inoculation, the SCN female numbers were counted. For each gene, at least ten plants with transgenic hairy roots were obtained and analyzed.

## Data Availability

The data presented in the study are deposited in the BioStudies database, accession number E-MTAB-12363.
