# Supplementary figures and images for: Comparisons of constitutive resistances to soybean cyst nematode between PI 88788- and Peking-type sources of resistance in soybean by transcriptomic and metabolomic profilings
Source: Front Genet. 2022 Nov 10;13:1055867. doi: 10.3389/fgene.2022.1055867 (PMC9686325; doi:10.3389/fgene.2022.1055867)

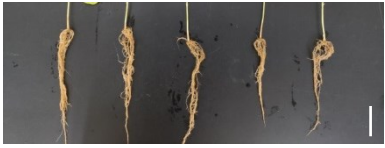

**CK**

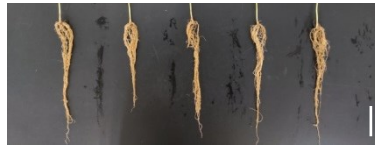

***GmERF71* OE**

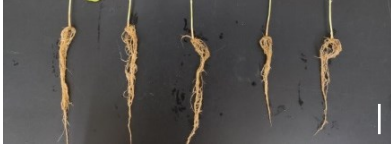

**CK**

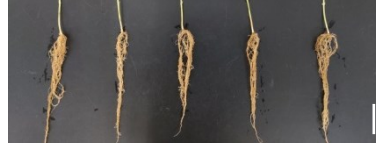

***GmLOX1* OE**

Bars=5cm

**Figure S5**

Supplement: Supplementary file 2 [file Image5.pdf]

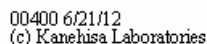

2Down: specifically down-regulated genes in PI 88788-type sources including Glyma.06G235900 (2.6.1.5).

Supplement: Supplementary file 7 [file Image4.pdf]
