# Supplementary material for: Comparisons of constitutive resistances to soybean cyst nematode between PI 88788- and Peking-type sources of resistance in soybean by transcriptomic and metabolomic profilings
Source: Front Genet. 2022 Nov 10;13:1055867. doi: 10.3389/fgene.2022.1055867 (PMC9686325; doi:10.3389/fgene.2022.1055867)
Supplement: Supplementary file 8 [file Image2.pdf]

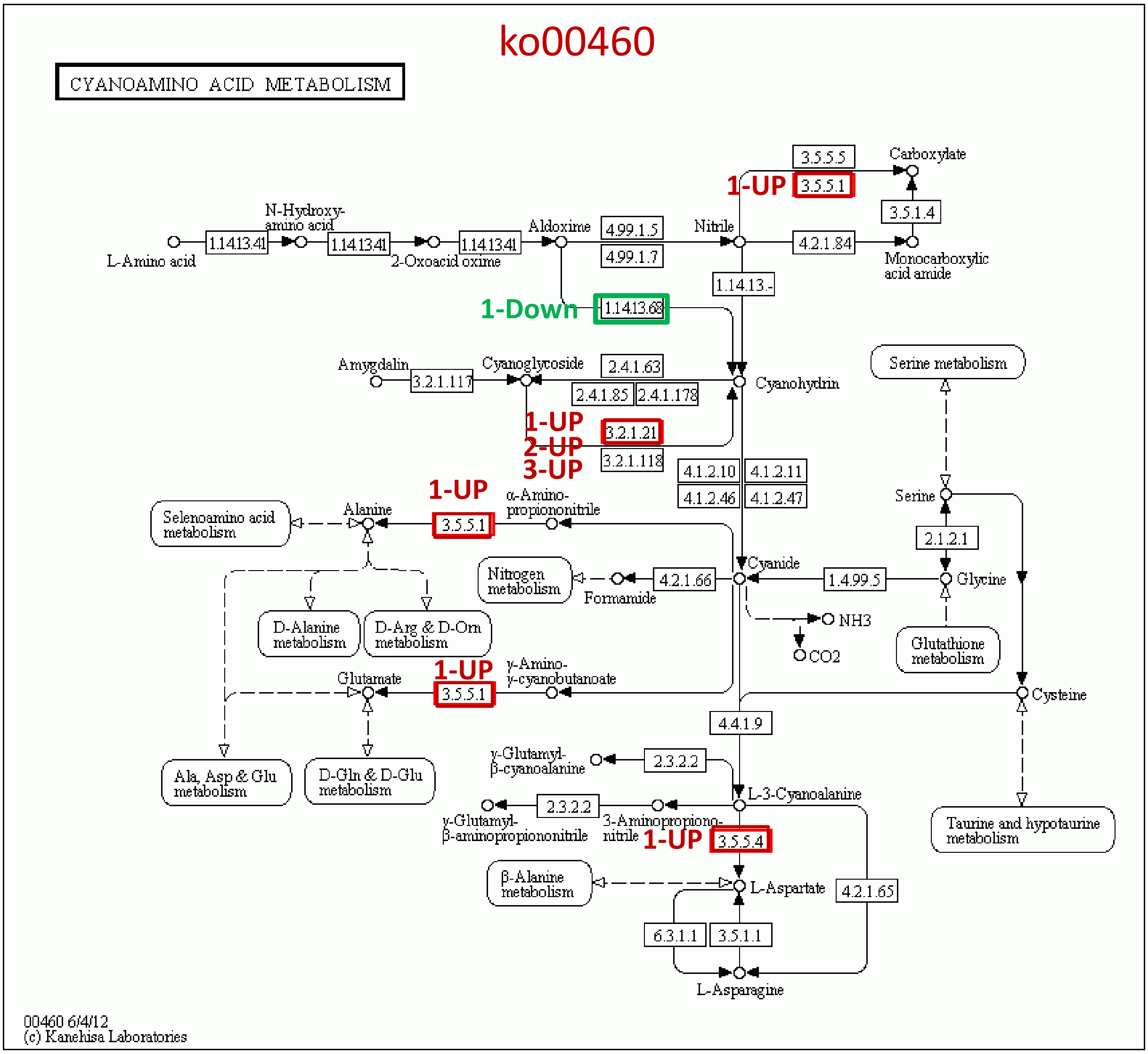

Figure S2

1UP: specifically up-regulated genes in Peking-type sources including Glyma.15G031400 (3.2.1.21), and Glyma.11G180900 (3.5.5.1 and 3.5.5.4).
2UP: specifically up-regulated genes in PI 88788-type sources including Glyma.15G270900 (3.2.1.21).
3UP: commonly up-regulated genes in both Peking- and PI 88788- type sources including Glyma.08G150100 (3.2.1.21).
1Down: specifically down-regulated genes in Peking-type sources including Glyma.18G080400 (1.14.13.68).
