# Supplementary material for: Comparisons of constitutive resistances to soybean cyst nematode between PI 88788- and Peking-type sources of resistance in soybean by transcriptomic and metabolomic profilings
Source: Front Genet. 2022 Nov 10;13:1055867. doi: 10.3389/fgene.2022.1055867 (PMC9686325; doi:10.3389/fgene.2022.1055867)
Supplement: Supplementary file 10 [file Image3.pdf]

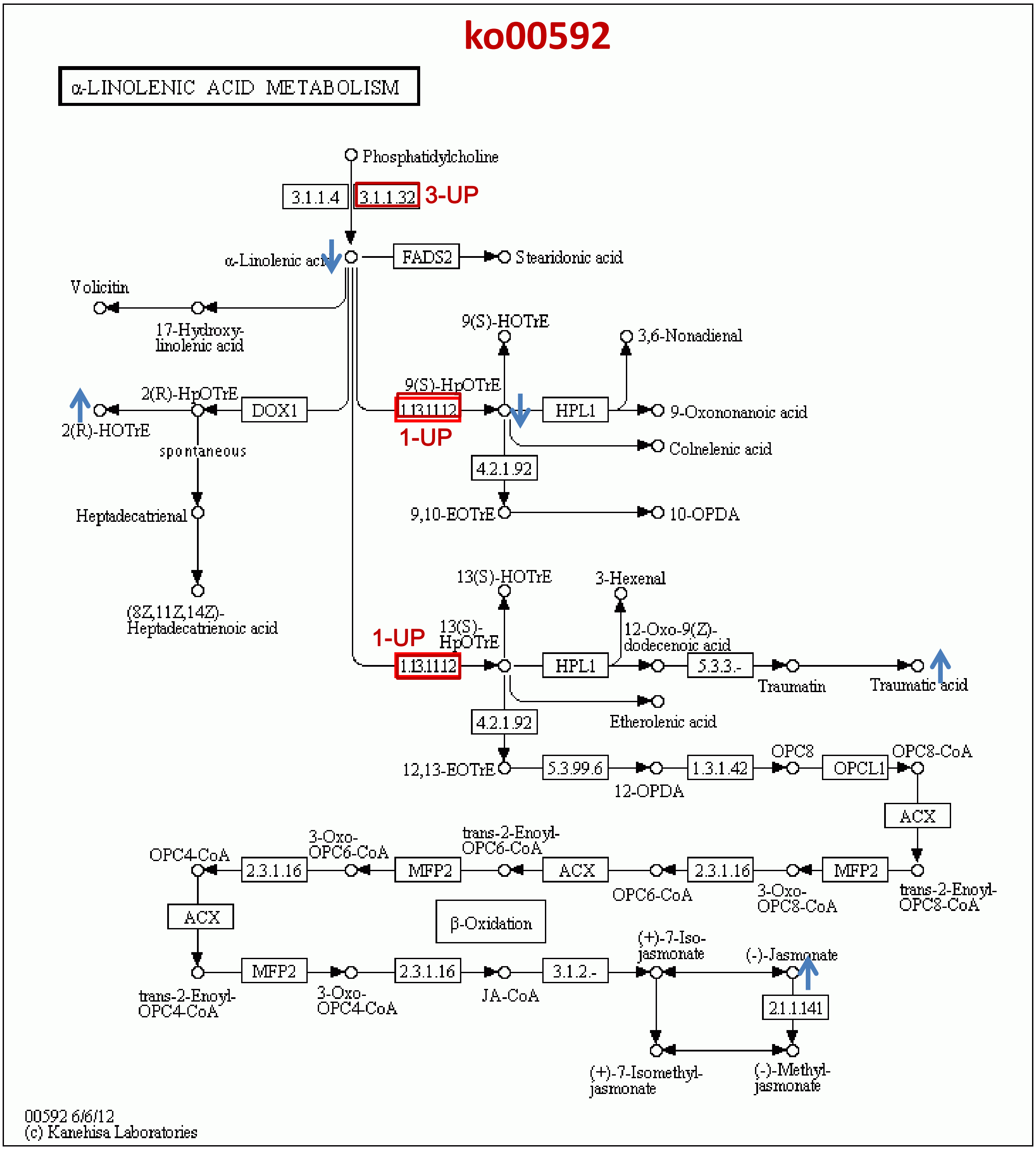

Figure S3

1UP: specifically up-regulated genes in Peking-type sources including Glyma.07G196800 (1.13.11.12).
3UP: commonly up-regulated genes in both Peking- and PI 88788- type sources including Glyma.07G204800 (3.1.1.32).
Upward and downward blue arrow represent the up- and down- regulated metabolites in the pathway.
