# Supplementary material for: Comparisons of constitutive resistances to soybean cyst nematode between PI 88788- and Peking-type sources of resistance in soybean by transcriptomic and metabolomic profilings
Source: Front Genet. 2022 Nov 10;13:1055867. doi: 10.3389/fgene.2022.1055867 (PMC9686325; doi:10.3389/fgene.2022.1055867)
Supplement: Supplementary file 15 [file Image1.pdf]

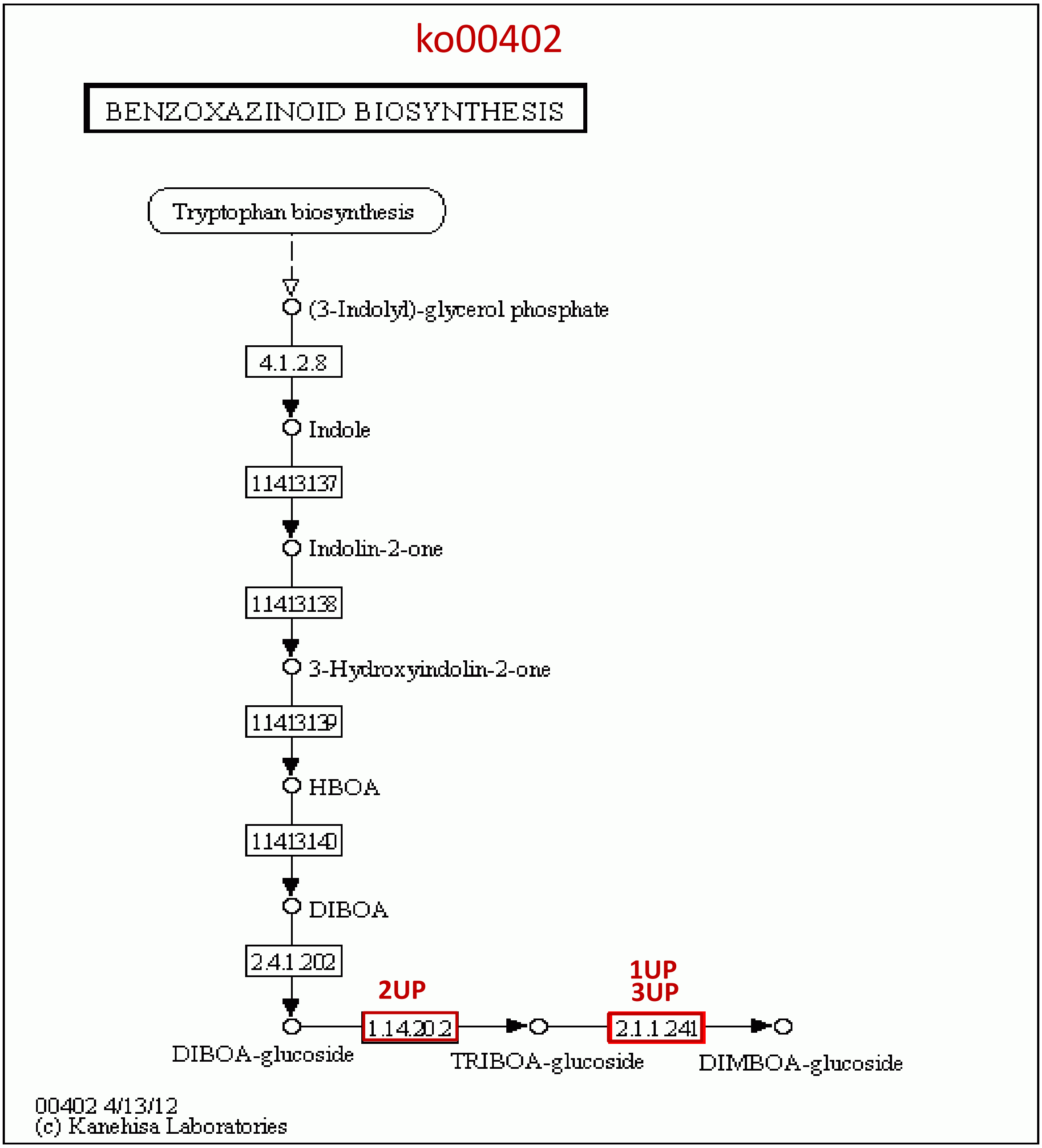

Figure S1

1UP: specifically up-regulated genes in Peking-type sources including Glyma.06G286600, Glyma.09G094600 and Glyma.06G286200 (2.1.1.241).
2UP: specifically up-regulated genes in PI 88788-type sources including Glyma.09G144900 and Glyma.09G145000 (1.14.20.2).
3UP: commonly up-regulated genes in both Peking- and PI 88788- type sources including Glyma.09G094400 (2.1.1241).
